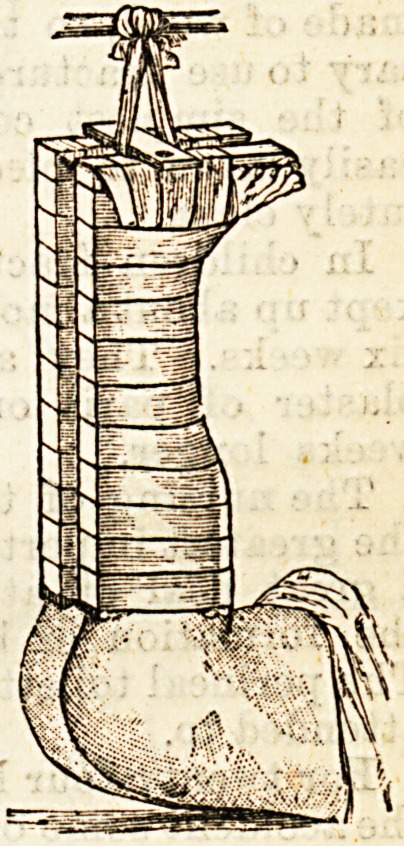# Treatment of Simple Fractures of the Femur

**Published:** 1893-04-29

**Authors:** 


### THE HOSPITAL CLINIC.

[*The Editor will be glad to receive offers of co-operation and contributions from members of the profession All letters should be addressed to* THE EDITOR, THE LODGE, PORCHESTER SQUARE, LONDON, W.]

### THE LEEDS GENERAL INFIRMARY.

### TREATMENT OF SIMPLE FRACTURES OF THE FEMUR.

In hospital practice, fractures of the femur form a very large class of the cases that are admitted for accident, and they are not peculiar to any period of life—fractures of the shaft occurring in quite young children, and the neck, unfortunately, not a very infrequent occurrence in old age—so they should receive the most careful consideration of surgeons, nurses, and students. We are afraid, in these days, there is a little too much running after the novel; and, in the interest attached to the latest surgical achievements, a subject like fractures, which the great surgeons of the past spent enormous time and labour in investigating, is apt to receive not quite the amount of attention the importance of it demands.

The femur is said to be one of the common bones in which non-union occurs, and unless great care is observed in the treatment, shortening, amounting to marked lameness, sometimes results.

In children, fracture of the shaft, about the middle, is the usual accident; these are sometimes of the green-stick variety, and if so, it may be some days after the accident before the patient is brought for treatment.

In Leeds, the invariable way of treating these fractures in children, is to fix them up in a Bryant's splint (*vide* diagram (507) "Bryant's Surgery," Vol. II.,

*India rubber Accumulator.*

page 445). This consists of two long splints, having an iron bracketed interruption opposite the hips; the splints extend from the axillæ, to six or eight inches below the soles of the feet; these are held together by means of two iron cross-bars, one going over the chest near the top of the splint, the other below the soles, so that a complete framework is made. These cross-bars can be made moveable by means of screws, so as to increase or diminish the distance between the two splints. On one of the splints near the lower end is a moveable foot-piece, to which is fixed the foot of the sound limb; on the outer side of the other splint is fixed an india-rubber accumulator, a pulley at the lower end, and another pulley on the lower cross-bar, from two to three inches from the inner side of the splint; the accumulator and pulleys are for making extension on the fractured limb

The patient is placed in this apparatus, great care being taken with the broken thigh. A stirrup made of a long piece of strapping, about the width of the leg, is fixed to both sides of the injured limb from the knee, making a loop below the foot, about three inches from the sole; in this loop is fixed a flat piece of wood with a hole in the middle. It must be wide enough to prevent pressure of the strapping on the malleoli. Theoretically the ends of the strapping should be fixed to the lower end of the femur, so as to get direct extension on the lower fragment, but this is not found to be of any real practical importance. The sound limb is now bandaged to the side having the foot piece, the fractured limb fixed to the other side, and a body bandage placed round the chest. Extension is now made on the fractured thigh by threading a piece of strong cord through the hole in the wooden stirrup and fastening it, passing the other end of the cord through the pulleys and fixing it to the elastic accumulator.

The child is now completely fixed in this excellent apparatus, and can be moved about, washed, and have other necessary attentions without disturbing the fracture.

The plan has sometimes been adopted of fixing up either one or both lower extremities at right angles to the trunk vertical extension, by means of a back splint with a foot-piece. The back-splint extends from the buttocks to the sole, and is suspended to something fixed over the bed. (See diagram 516, "Bryant's Surgery," vol. ii., p. 451.) By this method very young children can be easily attended to, and it is astonishing how comfortable they appear to be with their legs so suspended.

Fractures of the shaft in adults are sometimes treated in a Bryant's apparatus, but more often by means of a long splint, extending from the axilla to a few inches beyond the sole. A stirrup is fixed to the leg in the manner already described. The fracture having been reduced, the lower extremity is bandaged to the splint from the middle of the calf to just below the level of the fracture, and a body bandage round the upper part of the splint and the chest. A pulley is fixed to the foot of the bed, and weight extension is made by attaching to the cord from the stirrup some weights varying from 6 to 12 lbs., the cord going over the pulley at the end of the bed. The counter-extending force is procured by raising the foot of the bed some 8 or 10 inches, on wooden blocks, so that the weight of the body is pulling in opposite directions to the weights. Sometimes, in addition to the long splint, it is necessary to have three short splints around the femur.

This method gives excellent results, and, on the whole, is very comfortable.

The so-called extra-capsular fractures of the neck (those produced by direct violence) are treated in the same way. It is very important to manipulate these with the greatest care, as they are often impacted. By rough treatment the impaction may become loosened, an alteration not to be desired. (This remark also applies to the so-called intra-capsular fractures.) If they are not impacted, there is often a good deal of shortening; it may then be necessary to put a few additional pounds on the weight extension. The intra-capsular fractures are sometimes treated by the same method for two or three weeks, or the limb is simply placed between sand-bags. These fractures generally occur in old people, and it is important not to keep them for too long a time in the recumbent position, as they run great risks of getting hypo-static pneumonia, &c. We know also from clinical experience that these fractures, if not impacted rarely unite with bone, so that at the end of a fortnight or three weeks the patients should get up, the hip having been fixed either in starch or plaster of Paris, or a hip-splint of leather or poro-plastic material.

Transverse fractures just above the condyles are not common accidents, and are often difficult to diagnose. The knee is flexed, so that the upper end of the lower fragment projects into the popliteal space, and the lower end of the upper fragment rests on the anterior surface of the lower. In these cases an anæsthetic is generally necessary before the fracture can be reduced, and sometimes it is necessary and advisable to divide the Tendo Achillis. Having reduced the fracture, the lower extremity is placed on a double inclined plane. The same apparatus can be used for the treatment of separation of the lower epiphysis of the femur. This is not a common accident. The deformity is curious, as the epiphysis is displaced forwards, and the lower end of the diaphysis forms a prominence in the popliteal space. Certain fractures of the femur offer special difficulties to reduction, *e.g.*, the fracture below the lesser trochanter; here the upper fragment is flexed and rotated outwards, and the lower fragment pulled upwards and inwards. The ingenuity of the surgeon is often taxed, and special apparatus may have to be devised, such as Hodgen's splint, the double inclined plane, or some other modification.

In Leeds the majority of the beds are made of wood, so that it is not necessary to use fracture boards. They are of the simplest construction, can be easily taken to pieces and kept absolutely clean.

In children fractures are generally kept up about a month, in adults about six weeks. They are then put up in plaster of paris or starch for a few weeks longer.

The nursing of these patients is of the greatest importance. They require a great deal of attention to prevent the formation of bed or splint sores. The perineal toilette must be carefully attended to.

For twenty-four hours or more after the accident some of the patients suffer from retention of urine, more especially males. They are often shy, and will suffer a good deal of discomfort before calling attention to their condition. This must be enquired into, and the necessary relief given.

## Figures and Tables

**Figure f1:**
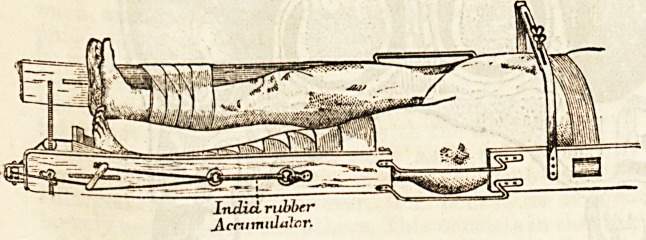


**Figure f2:**